# Risperidone-Loaded Nasal Thermosensitive Polymeric Micelles: Quality by Design-Based Formulation Study

**DOI:** 10.3390/pharmaceutics16060703

**Published:** 2024-05-24

**Authors:** Bence Sipos, Gábor Katona, Ildikó Csóka

**Affiliations:** Institute of Pharmaceutical Technology and Regulatory Affairs, Faculty of Pharmacy, University of Szeged, Eötvös Street 6, H-6720 Szeged, Hungary; katona.gabor@szte.hu (G.K.); csoka.ildiko@szte.hu (I.C.)

**Keywords:** polymeric micelle, nasal delivery, risperidone, quality by design, factorial design, quality control

## Abstract

The current research aims to develop thermosensitive polymeric micelles loaded with risperidone for nasal administration, emphasizing the added benefits of their thermosensitive behavior under nasal conditions. An initial risk assessment facilitated the advanced development process, confirming that the key indicators of thermosensitivity were suitable for nasal application. The polymeric micelles exhibited an average size of 118.4 ± 3.1 nm at ambient temperature and a size of 20.47 ± 1.2 nm at 36.5 °C, in both cases in monodisperse distribution. Factors such as pH and viscosity did not significantly impact these parameters, demonstrating appropriate nasal applicability. The model formulations showed a rapid, burst-like drug release profile in vitro, accompanied by a quick and high permeation rate at nasal conditions. Overall, the Quality by Design-based risk assessment process led to the development of an advanced drug delivery system capable of administering risperidone through the nasal cavity.

## 1. Introduction

Enhancement of bioavailability regarding central nervous system-associated drugs is of paramount importance and is one of the main topics of focus in current technological and clinical research and development trends [1,2,3]. Out of the utilizable techniques, nanotechnology offers many solutions to improve water solubility, thus improving the drug release and permeability profile of active substances with poor pharmacokinetics [4,5]. The decreased particle size is a promising feature but besides that, stable, biodegradable formulations must be found and developed in order to fully achieve a value-added formulation [6].

Polymeric micelles are colloidal nanocarriers with a typical size range of 10 to 200 nm depending on their polymeric structure and surface decorations [7,8,9]. Their building block is the so-called amphiphilic block. Co-polymers have a huge versatility and, most importantly, they are more stable in biological media and are biodegradable and biocompatible [10]. This versatility also offers the potential for the development of intelligent polymer-based systems, such as thermosensitive polymeric micelles. Besides the critical micellar concentration (CMC) and critical micellar temperature (CMT), a third important physicochemical value, the low critical solution temperature (LCST), characterizes them [11,12]. At lower temperatures, they are variable in size and size distribution but upon heating and reaching the LCST, they can decrease in size in a uniform manner. This is caused by the mechanical distortions in the micellar corona. Simultaneously, the encapsulated active substances can be released in a rapid manner due to the infiltration of water into the micellar core. This mechanism can be exploited in multiple administration routes and the rapid drug release could lead to rapid drug permeation [13,14,15].

Nasal drug delivery is one of these administration routes, where rapid drug release is extremely important, if not the most important due to the highly paced mucociliary clearance, eliminating most of the administered drugs in about 15 to 20 min. On the other hand, the highly vascularized nasal mucosa allows rapid absorption of the blood via the nose-to-blood pathway [16,17,18]. The nose-to-brain pathway is also auspicious due to the fact that the axonal transport in the olfactory region through the olfactory and trigeminal nerves bypasses the blood–brain barrier (BBB). Thus, central nervous system-associated diseases are of paramount importance where most of the drugs have limited access to the brain [19,20]. Risperidone, our model drug applied in this study, can be utilized for the treatment of schizophrenia and bipolar depression-related acute mania. Risperidone is a Biopharmaceutical Classification Class II drug, with low water solubility limiting its efficacy [21]. RIS was chosen as our model drug due to its lipophilicity, suitable molecular weight for nasal administration (410.05 Da), and therapeutic potential via the nasal administration route. Due to the restrictions based on the low administrable volumes (25–200 µL), a concentrated dosage form with solubilized RIS is desired for this administration route. The nose-to-brain pathway can also be beneficial in this case, as the target site is easily accessible. Acute mania is treated generally with intravenous injections or orally dispersed tablets; however, many therapeutic protocols in other acute states also prefer the nasal administration route. For example, in the case of opioid toxication, nasal naloxone is administered, and, for diabetes-related hypoglycemia, glucagon is applied this way [22,23].

Risperidone was utilized as a model active agent in order to increase its bioavailability via the nasal administration route. Kumar et al. prepared mucoadhesive nanoemulsions to satisfy this goal. Based on their findings, a quick and effective brain transport was evaluated due to the decrease in particle size and the encapsulation of RIS into the nanocarrier system [24]. As another type of lipid nanocarrier system, solid lipid nanoparticles (SLNs) were developed by Patel et al., indicating the superiority of the RIS-loaded SLNs compared to the intravenous reference. A quick brain uptake was detected via in vivo animal studies 1 h post-administration reflecting on a rapid absorption profile. This 1 h period is significantly better compared to currently utilized formulations [25]. Liposomes are also of interest in current drug research and development processes as promising nanocarriers. Narayan et al. reported a formulation of RIS encapsulated in PEGylated liposomes designed for nasal administration. The in vivo study also showed the superiority of intranasal administration compared to intravenous bolus administration of pure drugs [26]. The encapsulation of RIS in polymeric micelles designed for nasal administration has not yet been reported to the best of our knowledge. The main advantage of encapsulation of RIS in polymeric micelles over lipid- and protein-based drug delivery is the higher solubilizing efficiency, leading to higher drug loading and water solubility increase. Polymeric micelles are also considered more stable in the biological milieu and respond against varying conditions more efficiently towards burst-like drug release or circulation stability, depending on the composition.

Exploiting the advantages of the nasal administration and the thermosensitive nature of the nanocarriers, our aim was to perform factorial-design- and quality-control-based development of risperidone-loaded polymeric micelles for nasal drug delivery. As an additive solubilizer, the effect of Pluronic^®^ F-108 on the thermosensitive nature of Pluronic^®^ F-127 was investigated based on a quality-controlled development environment using risk assessment processes. The aim for the optimized product is to have a versatile applicability range at various conditions, most specifically on the conditions of the nasal cavity, alongside the capability to provide rapid, burst-like drug release required for this specific route. As a future possibility, this prototype formulation could be an advantageous alternative in the therapy of acute mania outbursts in clinical care.

## 2. Materials and Methods

### 2.1. Materials

Pluronic^®^ F-108 (F108) (PEG_136_-PPG_52_-PEG_136_ (poly(ethylene-glycol)–*block*–poly(propylene-glycol)–*block*–poly(ethylene glycol); average molecular weight: 14,600 Da); Pluronic^®^ F-127 (F127) (PEG_95_-PPG_62_-PEG_95_ (poly(ethylene-glycol)–*block*–poly(propylene-glycol)–*block*–poly(ethylene glycol); average molecular weight: 12,500 Da) and risperidone (RIS; 3-[2-[4-(6-fluoro-1,2-benzisoxazol-3-yl)-1-piperidinyl]ethyl]-6,7,8,9-tetrahydro-2-methyl-4H-pyrido[1,2-a]pyrimidin-4-one; water solubility: <0.1 mg/mL; logP: 2.7; molecular weight: 410.49 g/mol; oral bioavailability: 70%) were acquired from Sigma-Aldrich Co., Ltd. (Budapest, Hungary). Chitosan (Chit, <100 kDa), sodium hyaluronate (HyA, low molecular weight, 20–40 kDa), and hydroxypropyl methylcellulose (HPMC, average molecular weight: 10 kDa, viscosity of 80–120 cP, 2% in water (20 °C)) were also acquired from Sigma-Aldrich Co., Ltd. Sodium hydroxide and 1 n hydrochloric acid were used for pH setting. For the in vitro nasal investigations, Simulated Nasal Electrolyte Solution (SNES) was utilized with the following composition: 2.98 g/L of potassium chloride, 8.77 g/L of sodium chloride, and 0.59 g/L of anhydrous calcium chloride in 1000 mL of purified water, adjusted to a pH of 5.6 [27].

### 2.2. Initial Risk Assessment on Nasally Applicable Thermosensitive Polymeric Micelles

Quality by Design-based risk assessment was performed via the extended R&D methodology for the early phases of preclinical studies [28,29,30]. At first, the Quality Target Product Profile was set up, followed by the determination of Critical Quality Attributes (CQAs), Critical Process Parameters (CPPs), and Critical Material Attributes (CMAs). Based on the developed knowledge space, a two-level risk assessment was executed using the LeanQbD^®^ Software 4.0 (QbD Works LLC, Fremont, CA, USA). At first, an interdependence rating was performed where the relations between the QTPP–CQA and CQA–CPP/CMA elements were assigned on a 3-level scale. The scale was as follows: low–medium–high influential relations to each other. To quantify the severity of the risk factors, the probability of the influencing factors was also determined, and using the software Pareto, diagrams were set up based on the calculated severity scores [31].

### 2.3. Quantitative Analysis of Risperidone via High-Performance Liquid Chromatography

The quantification of RIS was performed with high-performance liquid chromatography (HPLC) using an Agilent Infinity instrument (Agilent Technologies, Santa Clara, CA, USA). As a stationary phase, a Kinetex^®^ C18 column (5 µm, 150 mm × 4.6 mm (Phenomenex, Torrance, CA, USA)) was used. The injection volume was 10 µL and the temperature was set at 25.0 °C. As mobile phases, acidic purified water (0.1% *w*/*v* formic acid) (A) and acidic acetonitrile (0.1% *w*/*v* formic acid) (B) were applied. The separation was performed with isocratic elution for 6 min. Chromatograms were detected at 280 ± 4 nm using a UV–Vis diode array detector. The retention time was 2.65 min. The chromatograms were evaluated using ChemStation B.04.03. Software (Agilent Technologies, Santa Clara, CA, USA). The limit of detection (LOD) and limit of quantification (LOQ) of RIS were 4.78 and 15.75 ppm, respectively. The calibration was performed between 5 to 100 µg/mL, where the determined coefficient of linearity (R^2^) value was 0.9999.

### 2.4. Characterization of the Blank Mixed Polymeric Micellar Composition

#### 2.4.1. Dynamic Light Scattering Measurements

Dynamic light scattering (DLS) measurements were performed via the Malvern Nano ZS Zetasizer (Malvern Instruments, Worcestershire, UK). At first, it was applied to determine the LCST temperature via the tracking of the change in average hydrodynamic diameter, corresponding to the micelle size [32,33,34]. At the LCST temperature, as mentioned before, the nanoparticles can increase in size followed by an extreme drop. Thus, this physicochemical change can be observed to detect LCST. A gradient temperature increase was utilized in the temperature range of 25.0 to 40.0 °C. The samples were placed in folded capillary cells and left inside during the entire measurement process. The average hydrodynamic diameter was registered at every 1 °C increment. Between each measurement, a 2 min lag time was provided for proper heating. To determine the micelle size and size distribution (expressed as polydispersity index, PdI), a refractive index of 1.335 was used.

#### 2.4.2. Effect of the Mixed Micellar Composition on the LCST Temperature

Blank (drug-free) solutions were prepared for the mixed micellar systems. An amount of 5 mg/mL of stock solutions of each co-polymer was prepared and mixed with the following F127: F108 ratios: 5:1, 4:1, 3:1, 2:1, 1:1, 1:2, 1:3, 1:4, and 1:5, respectively. The criterion was the following: the LCST temperature should be above ambient temperature (25.0 °C) and below the typical nasal temperature (36.5–37.0 °C). This temperature criteria provides that the burst-like drug release would not occur at ambient temperatures, but rather at the body’s conditions. All measurements were carried out in triplicate with individual batches (*n* = 3), and the results are expressed as the average ± SD.

#### 2.4.3. Effect of the Mixed Micellar Composition on Micelle Size and Distribution

After the determination of the LCST temperature of each composition, the micelle size and size distribution were investigated at the temperature of 36.5 °C. Each composition was taken into consideration at the mentioned ratios. All measurements were carried out in triplicate with individual batches (*n* = 3), and the results are expressed as the average ± SD.

#### 2.4.4. Characterizing Effect of Temperature on the Optimized Mixed Micellar Composition’s Micelle Size and Size Distribution

The optimal composition’s thermosensitive behavior was described by measuring the micelle size and size distribution in the temperature range of 25.0–40 °C. All measurements were carried out in triplicate with individual batches (*n* = 3), and the results are expressed as the average ± SD.

### 2.5. Optimization of the Drug Loading

To prepare RIS-loaded polymeric micelles, the direct freeze-drying method was utilized. Ethanolic solution of RIS was prepared and mixed with the aqueous co-solution of F127 and F108 at different ratios. An amount of 1.0 mL of the mixture was placed in vials and freeze-dried using a ScanVac CoolSafe 100–9 laboratory apparatus (LaboGene, ApS, Lynge, Denmark). The freezing was performed at −40 °C for 12 h under a 0.013 mbar pressure with an additional 6 h of secondary drying at 25 °C. The samples were dissolved with 1 mL of purified water. Freeze-drying is a crucial step in the formulation of nanoparticles, as it also increases their physical stability. Thus, the formulation was considered as a powder to be ex tempore dispersed in the proper liquid media prior to the administration.

To optimize the encapsulation of RIS into the mixed micellar system, a 2^3^-factorial design was set up. A 9-run design was set up, where the effect of 2 variables was investigated at 3 levels on micelle size and micelle size distribution (Table 1). As response variables, the micelle size and size distribution were selected.

The effect on micelle size and micelle size distribution was investigated after the reconstitution process, analyzing the quadratic response surface and constructing a second-order polynomial model using TIBCO Statistica^®^ 13.4 Software (Statsoft Hungary, Budapest, Hungary). The relationship of the variables can be described with the following second-order equation:

(1) $$Y=\beta_{0}+{\beta_{1}x}_{1}+\beta_{11}x_{1}^{2}-\beta_{2}x_{2}-\beta_{22}x_{2}^{2}$$

where Y is the response variable; β_0_ is a constant; β_1_ and β_2_ are linear coefficients; and β_11_ and β_22_ are quadratic coefficients. The analysis of variance (ANOVA) statistical analysis was carried out in harmony with the *p*-values, where a variable was considered significant if the *p*-value was less than 0.05 at a 95% confidence level.

### 2.6. Characterization of the Drug-Loaded Mixed Micellar Formulation

#### 2.6.1. Effect of Drug Loading on the Thermosensitive Nature of the Carrier

The optimized, freeze-dried RIS-loaded micellar formulation was dissolved in purified water and placed in folded capillary cells for the DLS measurements. The refractive index was set to 1.677. To determine whether drug encapsulation had an effect on the LCST, the micelle size and size distribution were measured in the range of 25.0–40.0 °C. All measurements were carried out in triplicate with individual batches (*n* = 3), and the results are expressed as the average ± SD.

#### 2.6.2. Effect of pH and Viscosity on the LCST Temperature of the Risperidone-Loaded Polymeric Micelles

To determine the effect of pH on various parameters, a solution series was prepared using sodium hydroxide and hydrochloric acid in the range of pH 1–14, with an increment of 1.0. A different solution series with varying viscosity was prepared via HPMC in the viscosity range of 5.0–40.0 cP, with an increment of 5.0.

These solutions were applied for the dissolution of each freeze-dried RIS-loaded polymeric micelle formulation. DLS measurements were performed in the range of 25.0–40.0 °C to determine the LCST. All measurements were carried out in triplicate with individual batches (*n* = 3), and the results are expressed as the average ± SD.

#### 2.6.3. Effect of pH and Viscosity on the Micelle Size and Size Distribution of the Risperidone-Loaded Polymeric Micelles

The same solution series were prepared as described before (Section 2.6.2) and the freeze-dried samples were dissolved in them. The micelle size and size distribution were registered via DLS at two different temperatures: 25.0 and 36.5 °C. All measurements were carried out in triplicate with individual batches (*n* = 3), and the results are expressed as the average ± SD.

#### 2.6.4. Effect of pH and Viscosity on the Thermodynamic Solubility of Risperidone-Loaded Polymeric Micelles

The thermodynamic solubility was measured via the saturation method. The applied varying pH and viscosity solution series were used for the dissolution of the freeze-dried samples (1 mL of each); however, the freeze-dried powders were placed on top of the stirring solutions until it was visibly determined that the system could not dissolve the solutions anymore. The system was kept stirred for 72 h (25 °C, 250 rpm) and covered with a parafilm. Then, the solutions were filtered through a 0.22 µm-pore-sized PES (polyether sulfone) membrane. The filtered RIS was measured via HPLC. All measurements were carried out in triplicate with individual batches (*n* = 3), and the results are expressed as the average ± SD.

#### 2.6.5. Effect of pH and Viscosity on the Encapsulation Efficiency of Risperidone-Loaded Polymeric Micelles

The encapsulation efficiency (EE%) was measured via the indirect method [31]. Samples were dissolved completely in the mentioned viscosity and pH solution series. The RIS-loaded micelles were separated from the aqueous media via centrifugation using a Hermle Z323 K high-performance refrigerated centrifuge (Hermle AG, Gosheim, Germany) at 14,000 rpm, 4 °C, for 45 min. The clear supernatant was diluted 5-fold with methanol, and then quantitative measurements were carried out via HPLC. All measurements were carried out in triplicate with individual batches (*n* = 3), and the results are expressed as the average ± SD. The encapsulation efficiency was calculated via this equation:

(2) $$EE\left(\%\right)=\frac{initial\;RIS\left(mg\right)-measured\;RIS\;in\;the\;supernatant(mg)}{initial\;RIS(mg)}\times 100$$

### 2.7. Validation of the Formulation at In Vitro Nasal Conditions

#### 2.7.1. Formulation of Model Preparations

For the validation of the nasal applicability of the RIS-loaded polymeric micelles, 4 different formulations were applied with a constant RIS amount of 5 mg. The first formulation was the pure RIS-loaded polymeric micelle dissolved in SNES at pH 5.6 (RIS-PM). The other formulation contained chitosan (RIS-PM_Chit) as a widely applied nasal mucoadhesive agent, with a concentration of 0.25% *w*/*v* [35]. To prepare RIS-PM_Chit, chitosan was dissolved in pH 5.0 purified water, and then this solution was used to dissolve the freeze-dried cakes. The third formulation contained hyaluronic acid (RIS-PM_HyA), in a concentration of 0.50% *w*/*v*, which is applied generally to sustain the release profile of drugs in the nasal cavity [32]. To prepare this sample, HyA was dissolved in pH 5.6 SNES at 4 °C, followed by the dissolution of the freeze-dried cakes. The fourth formulation was used as a reference; an RIS suspension (RIS_Susp) was prepared in pH 5.6 SNES with the addition of a low concentration of HyA (0.05% *w*/*v*) to prevent rapid sedimentation. The main physicochemical characteristics of the formulations can be seen in Table 2.

#### 2.7.2. In Vitro Drug Release Study

The drug release study was performed using the modified paddle method. The mentioned 4 formulations were placed in dialysis tubes (Spectra/Por^®^ Dialysis Membrane with a 3.5 kD MWCO value (Spectrum Laboratories Inc., Rancho Dominguez, CA, USA)). The tubes were placed in 100 mL of SNES as the dissolution media. The temperature was set at 36.5 °C and the paddle rotated at 100 rpm. At predetermined time points, 0.5 mL of aliquots were taken from the dissolution media for up to 60 min. The quantification of RIS in the dissolution media was performed via HPLC. All measurements were carried out in triplicate with individual batches (*n* = 3), and the results are expressed as the average ± SD.

#### 2.7.3. In Vitro Nasal Passive Diffusion Study

A modified Side-bi-Side^®^ horizontal diffusion cell was applied to determine the passive diffusion of RIS in the formulations. A regenerated cellulose membrane (Whatman™ (0.45 µm, 25 mm)) was impregnated with isopropyl myristate with a surface of 0.785 cm^2^ and used as the diffusion barrier between the acceptor and donor compartments. Both compartments’ volumes were 9.0 mL and the temperature was set at 36.5 °C. The donor phase consisted of SNES and the acceptor phase was a pH 7.4 PBS. At predetermined time points, 50 µL of aliquots were taken from the acceptor chamber, and then concentration was measured via HPLC. The taken aliquots were immediately replaced with the same volume of pH 7.4 PBS. Flux (J), as the cumulative permeability per time point, was calculated via the following equation:

(3) $$J=\frac{m_{t}}{A\times t}$$

where *m_t_* is the permeated drug amount at *t* time and *A* is the surface of the membrane.

## 3. Results

### 3.1. Initial Risk Assessment

The initial risk assessment served the purpose of gathering all relevant information regarding the applied specific co-polymers and their possible effect on nasal administration to determine which factors influence the performance of the formulation the most. At first, the QTPP elements were set up. The QTPP elements were selected based on the ICH Q8 guideline with the addition of the nanocarrier system. The main goal was to provide a liquid nasal dosage form, with the additional value of rapid drug release and permeation triggered by the phase transition above the LCST. The dosage form should be a solution, due to the encapsulation of the poorly water-soluble model active substance, risperidone. The nanocarrier criteria can be divided into two conditions. First, the polymeric micelles should be in the micelle size range of 10 to 200 nm with a monodisperse distribution. Second, the thermoresponsive behavior should undergo nasal conditions. Regarding indication and target population, the idea behind this subject is the possibility of reaching the central nervous system more efficiently, most specifically in the adult population. The drug release and absorption profile should be quick due to the limited residence time in the nasal cavity. The QTPP elements can be summarized in Table 3.

**Figure 1:**
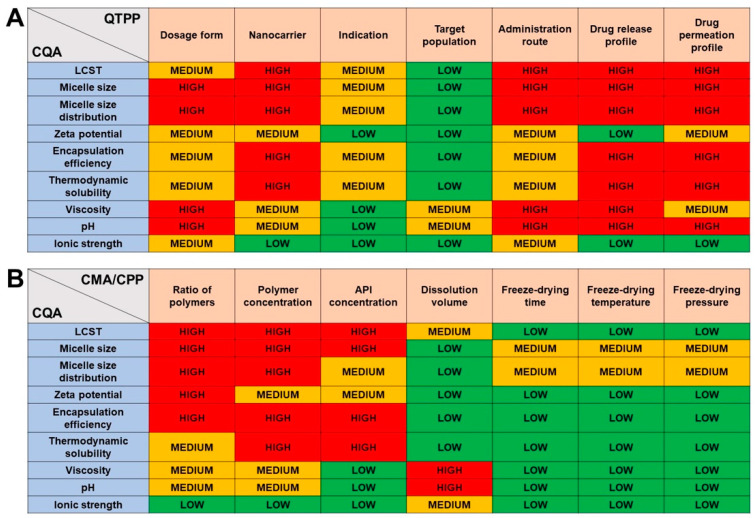
Interdependence rating amongst CQA–QTPP (**A**) and CQA–CMA/CPP elements (**B**).

The risk assessment was performed at two levels. At first, an interdependence rating was performed for which the results can be seen in Figure 1. The interdependence rating provided insight into the specific conditions of this formulation and QTPP elements and described how severely each factor influences the other.

**Figure 2:**
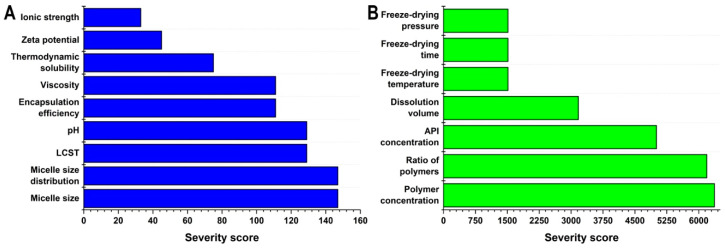
Result of the risk assessment process. The severity scores of each Critical Quality Attribute (**A**) and Critical Material Attribute/Critical Process Parameter (**B**).

To quantify the severity of the assigned relations, probability-based calculations were performed using the LeanQbD software. As a result, the severity score of each CQA and CMA/CPP element was obtained as seen in Figure 2.

The results indicate the following: Micelle size and size distribution greatly affect the target profile, due to the fact that water solubility enhancement is corroborated by the decrease in particle size. Uniform drug absorption is also considered to be important since it can only be obtained where the particles are monodispersely distributed. LCST plays a great role in fulfilling the target product profile since the rapid drug release due to the distortion of the hydrophilic corona must undergo nasal conditions. Zeta potential does not play a severe role in the case of our formulation since both co-polymers applied are non-ionic in nature. Polymeric micelles with almost zero surface charge can be absorbed quickly via paracellular transport across the nasal mucosa, thus the less influence on product quality. The material attributes prevailed in the case of the risk assessment of CMA and CPP factors. The applied polymers are thermostable at lower temperatures, thus additional risk can be omitted from the direct freeze-drying method.

Based on the results of the risk assessment, the following was taken into further consideration: (1) The LCST must be optimized prior to drug loading and blank mixed polymeric micelles must fit the criteria; (2) after finding the proper composition with the blank micelles, the drug loading must be optimized accordingly, where the polymeric concentration and the API concentration must be taken into account regarding the micelle size and distribution; (3) the effect of drug loading must be investigated prior to alternating the dissolution media; and (4) the pH and viscosity prevailed as critical factors compared to ionic strength, thus the effect of these variables must be investigated on all nanoparticle characteristics and also on the parameters describing the solubilization: encapsulation efficiency and thermodynamic solubility.

### 3.2. Characterization of Blank Mixed Micellar System

**Figure 3:**
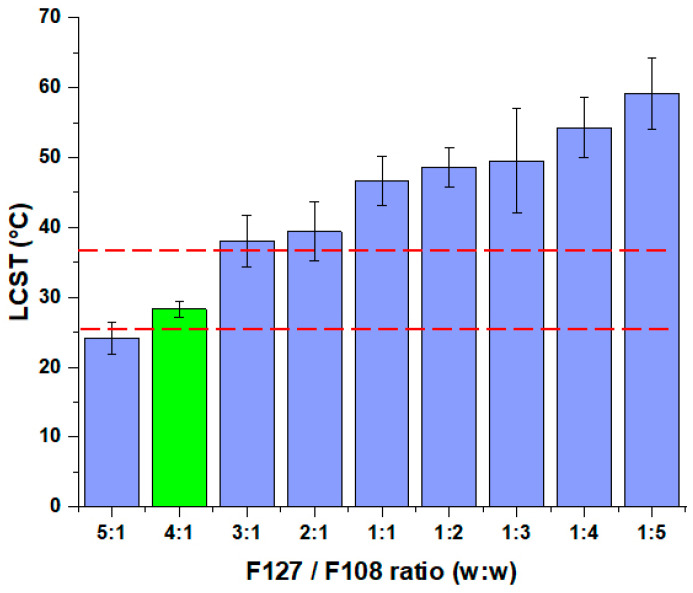
Determination of LCST via dynamic light scattering measurements. The decrease in micelle size was registered upon the increase in temperature from 25 to 40 °C. The red lines indicate the proper range for thermosensitive systems regarding the LCST value. Data are presented as means ± SD (*n* = 3).

At first, the optimal F127 to F108 ratio had to be determined based on the LCST and the micelle size and distribution. The results from the DLS measurements can be seen in Figure 3.

**Figure 4:**
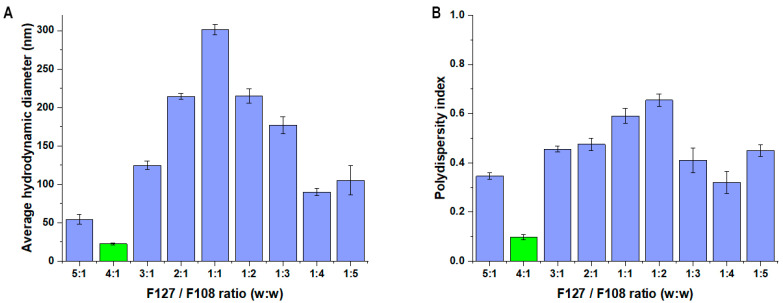
Micelle size (expressed as average hydrodynamic diameter) (**A**) and micelle size distribution (expressed as polydispersity index) (**B**) at 36.5 °C. Data are presented as means ± SD (*n* = 3).

Based on the LCST measurements, only one composition fits the criterion, which is that the LCST should be above ambient temperature (25 °C) and below the general nasal cavity temperature (36.5 °C). It can be seen that via the decrease in the F127 portion, an increase in the LCST can be experienced. This proves that F108 itself is not capable of thermoresponsive behavior and requires a larger portion of the F127 co-polymer. Despite this, the combined solubilizing effect can be exploited with the utilization of multiple polymers in a mixed micellar system. The next step was to determine the micelle size at 36.5 °C of each polymeric formulation. The result can be seen in Figure 4.

**Figure 5:**
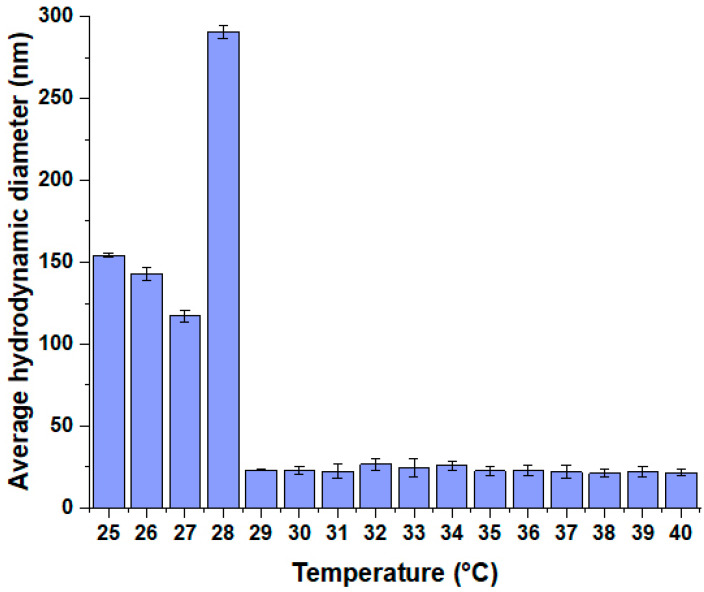
Change in micelle size (expressed as average hydrodynamic diameter) in the temperature interval of 25–40 °C. Data are presented as means ± SD (*n* = 3).

The only composition was the 4:1 in this case as well, where the smallest particle size could be experienced with monodisperse distribution. Regarding the 5:1 ratio, the particle size is also acceptable as it is considered lower; however, its polydispersity index was above 0.300, which means it has a polydisperse size distribution. Based on these results, the 4:1 ratio was investigated later on. The registration of micelle size can be seen in Figure 5 regarding the 4:1 F127 to F108 ratio.

The micelle size before the LCST was higher in monodisperse distribution compared to after the LCST. Mixed polymeric micelles generally tend to have higher micelle sizes compared to each of their own solutions above the critical micellar concentration. At a temperature of approximately 28 °C, a size increase can be observed. This phenomenon is typical for polymeric micelles, where at the phase transition temperature or at the critical micellar concentration, the particle size increases while the new thermodynamic stability-providing structure forms.

### 3.3. Optimization of RIS-Loaded Polymeric Micelles

The optimization was performed by a 2^3^-factorial design, where the effect of the amount of RIS and the combined amount of the micelle-forming polymers at a 4:1 ratio was investigated on micelle size and size distribution. The results from the factorial design can be seen in Table 4.

The results indicate that the formulation tends to be optimal where a low dose of RIS is applied. The higher polymeric concentration did not lead to the encapsulation of higher RIS dosage at this reconstitution volume (5 mL). The utilization of too much polymer is also not favorable since it would not dissolve rapidly or only partly in the dissolution media. Based on the results in Table 3, polynomial equations (Equations (4) and (5)) were constructed.

(4) $$Micelle\;size\left(nm\right)=132.20+111.32x_{1}-1.22x_{1}^{2}-22.42x_{2}+4.39x_{2}^{2}$$

The regression coefficient (R^2^) and adjusted regression coefficients (R^2^_adj_) of the surface plot were 0.9957 and 0.9897, respectively, indicating a good correlation. The positive coefficients indicate that by increasing the amount of these factors, the micelle size would also increase. The only significant (*p* < 0.05) factor was the amount of RIS (x_1_), proving the previous statement that a low-dose formulation is preferred.

(5) $$Polydispersity\;index=0.315+0.106x_{1}+0.000083x_{1}^{2}-0.037x_{2}-0.010x_{2}^{2}$$

**Figure 6:**
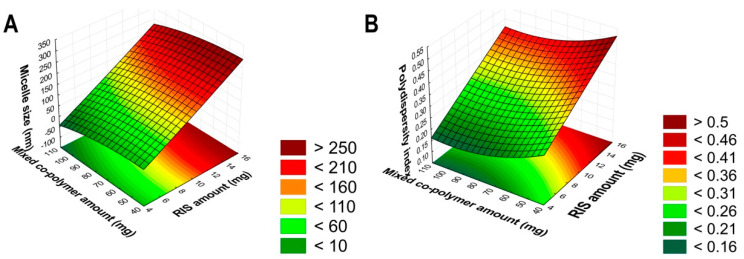
The 3D surface plots of the factorial design. The effect of the independent factors on micelle size (**A**) and micelle size distribution (polydispersity index) (**B**).

The R^2^ and R^2^_adj_ of this surface plot were 0.9914 and 0.9769, respectively, which also indicates proper correlation. In this case, the same can be claimed, in that only the amount of RIS (x_1_) is significant (*p* < 0.05) and by increasing this amount, the formulation would be polydisperse. The 3D-constructed surface plots can be seen in Figure 6.

Based on the results of the factorial design, Run No. 3 ought to be optimal in comparison with the surface plots and the generated equations. Thus, the formulation was prepared via the encapsulation of 5 mg of RIS in 80 mg of P-127 and 20 mg of P-108 with the freeze-drying method described previously.

### 3.4. Characterizing the Effect of Drug Loading on the Mixed Micellar System

To characterize the effect of drug loading, multiple parameters had to be checked. At first, the LCST of the drug-loaded polymeric micelle was investigated. The LCST of the system remained at 28 °C, indicating that the drug loading had not changed the thermosensitive behavior of the mixed polymeric micelle. The micelle size at ambient temperature was 118.4 ± 3.1 with a polydispersity index of 0.315 ± 0.009. At the temperature of the nasal cavity (36.5 °C), this size decreased to 20.47 ± 1.2 in monodisperse distribution, with a polydispersity index of 0.096 ± 0.014.

### 3.5. Effect of pH and Viscosity on the Mixed Micellar System

As critical applicability factors, the effect of pH and viscosity was investigated on the micelle characteristics. At first, the LCST temperature was determined (Table 5). The LCST was not affected by the acidic or alkaline conditions nor in the case of the viscosity change. A typical nasal liquid formulation’s pH is between 5.6 to 7.4, which matches the conditions of the nasal cavity; therefore, the formulation can perform its thermoreponsive behavior at these conditions. The same can be claimed for the viscosity, as commercially available, non-gel-forming preparations are also in the investigated range.

**Figure 7:**
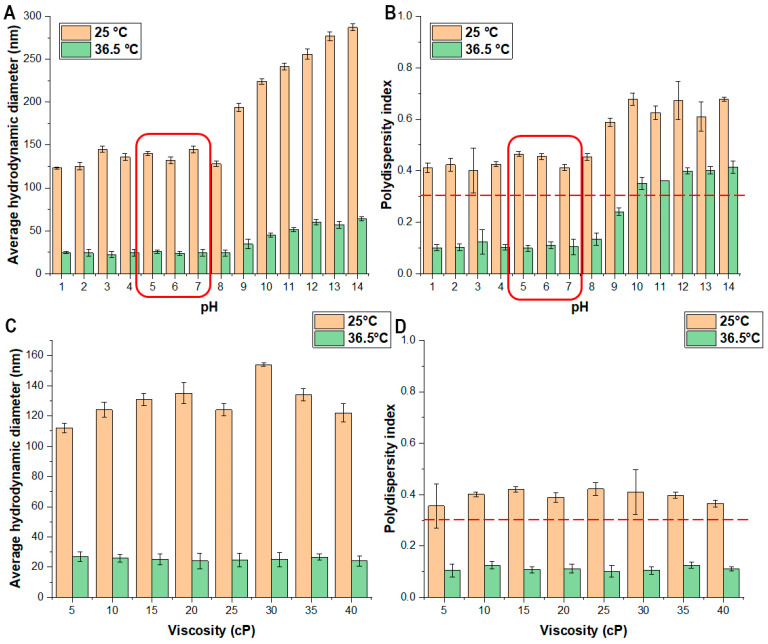
The effect of pH on the micelle size (**A**) and the micelle size distribution (**B**). The effect of viscosity on the micelle size (**C**) and the micelle size distribution (**D**). Data are presented as means ± SD (*n* = 3).

The next step was to determine the effect of pH and viscosity on the micelle size and the micelle size distribution. The results can be seen in Figure 7.

**Figure 8:**
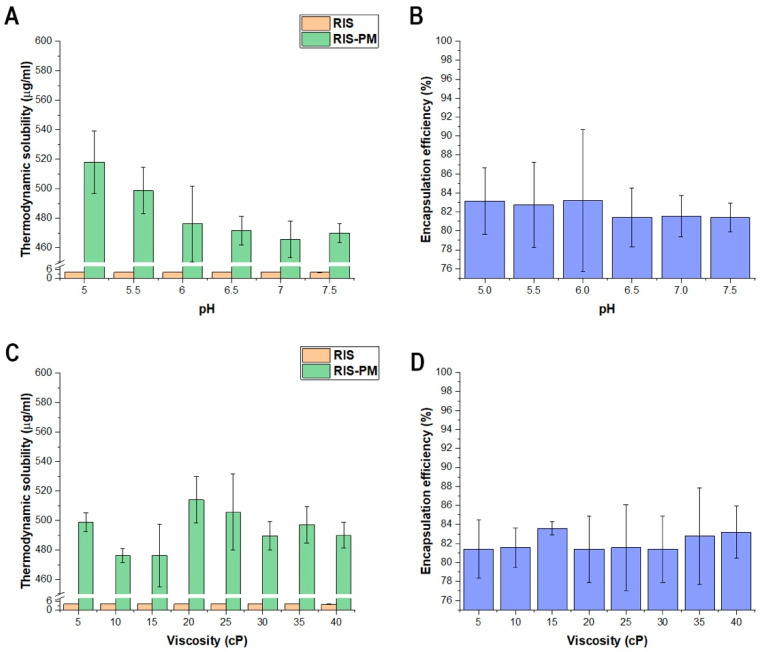
The effect of pH on thermodynamic solubility (**A**), on encapsulation efficiency (**B**), and the effect of viscosity on thermodynamic solubility (**C**) and encapsulation efficiency (**D**). Data are presented as means ± SD (*n* = 3).

Based on the results in Figure 8, it can be concluded that the pH influences the micelle size and distribution. In the pH of the nasal cavity, the formulation met the proper criteria of nasally applicable polymeric micelles; however, above pH 8.0, an increase can be experienced in both cases. Despite the fact that it did not change the LCST (Table 5), the Quality by Design-thinking allowed a thorough investigation with the conclusion that this polymeric micelle formulation cannot be applied at alkaline conditions since it does hold its proper nanoparticle characteristics. The increase can be explained by the degradation of polymers above a specific pH. The polymer degradation is supposed to be incomplete since it holds the LCST. The viscosity did not affect the micelle size and distribution.

The determination of encapsulation efficiency and thermodynamic solubility was performed between pH 5.0 and 7.5 since the previous results proved that the nanoparticle characteristics are only stable up to pH 8. The results can be seen in Figure 8.

**Figure 9:**
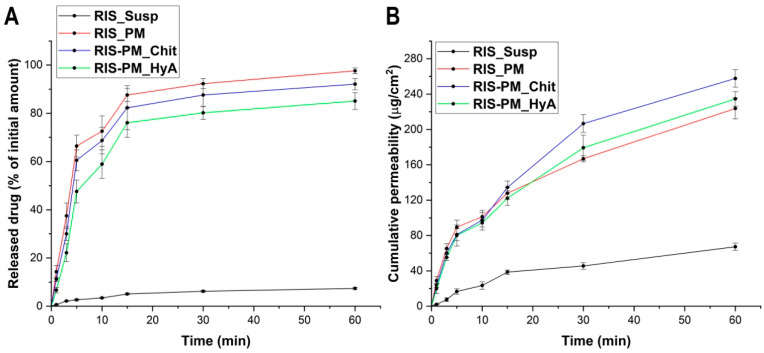
In vitro drug release study (**A**) and in vitro drug permeation study (**B**) with the model formulations compared to the initial RIS. Both measurements took place at simulated nasal conditions. Data are presented as means ± SD (*n* = 3).

A significant increase in thermodynamic solubility can be experienced at all pH and viscosity values compared to the barely soluble initial RIS (***, *p* < 0.0001). A slight decrease in the solubility increase can be seen in Figure 9A, which corroborates the previous statement that via the increase in pH, the nanoparticles are of less quality for nasal administration. Despite this, the encapsulation efficiency remained at all measured conditions. Moreover, the viscosity did not have a significant effect on the measured parameters.

### 3.6. Validation of the Formulation at In Vitro Nasal Conditions

To validate the optimal micelle composition, in vitro drug release and drug permeation studies were conducted. The results of these studies can be seen in Figure 9.

Based on the results of the in vitro studies, it can be observed that both the drug release and the drug permeation profile of RIS were enhanced after successful encapsulation into the polymeric micellar core. The binary system helped to achieve a rapid, burst-like drug release, which is desirable regarding nasal drug delivery systems. The individual differences regarding the composition did not significantly (*p* > 0.05) affect the release profile compared to each other. The same result was found in the drug permeation studies. Even though it did not significantly compare to the others, the chitosan-containing formulation exerted its permeability enhancer additive value.

## 4. Discussion

The current study focused on the quality-controlled development process and base investigations of advanced nanocarriers, categorized as one of the non-biological complex drugs. Risperidone, the model active substance investigated in this study, has potential in the treatment of acute mania disease related to bipolar depression, schizophrenia, and other psychotic illnesses. Currently, commercial products are used continuously to prevent this type of acute distress; however, an immediate relief solution has not been previously employed. With the increase in the prevalence of psychiatric diseases, it is of paramount importance to provide highly efficient drugs suitable for enhancing the overall brain bioavailability of the active substances.

The factorial design-based optimization came after a Quality by Design-associated risk assessment process, where the product quality criteria included the main advantages of thermosensitive polymeric micelles. The LCST temperature of the optimized mixed polymeric composition is adequate to distinguish the storage and the administration conditions (28 °C), as it is high enough to prevent the drug release at ambient temperature but low enough that the transition only occurs at the body’s temperature conditions, most specifically, at nasal conditions. The resulting small particle size was also confirmed after the drug loading process, with a micelle size of 20.27 nm in monodisperse distribution, with a PdI value of 0.096.

The micellar size can be influenced via various internal and external conditions, such as the two most crucial and investigated ones: the pH and the viscosity of the formulation [10,36,37,38]. Successful versatility was observed, as the formulation’s micellar size and distribution were not affected by the nasal conditions. This is also corroborated by the high thermodynamic water solubility and encapsulation efficiency (>80%). In comparison with prior studies, Pluronic^®^ F127 was rarely applied as a sole pharmaceutical excipient, thus Pluronic^®^ F108 was also included in this study as an additive solubilizer. The advantageous effect of Pluronic^®^ F127 has been proved before on chitosan microparticles and hydrogel formation could occur at much higher concentrations, which would also prolong the residence time in the nasal cavity [39,40]. In this study, a successful optimization was confirmed as the main focus was on the sole application of Pluronic F127.

Validation was performed via in vitro nasal drug release and permeation studies, where additional excipients were added at their typical concentration. A rapid, burst-like drug release was observed, followed by highly efficient and rapid drug permeation. Passive diffusion is a typical mechanism for nanocarriers and non-ionic polymeric micelles as well. This indicates that the formulation permeates quickly, which is a prosperous tactic against the elimination processes in the nasal cavity, the mucociliary clearance.

## 5. Conclusions

In conclusion, it can be claimed that the quality-controlled drug research and development strategy applied in this study prevailed. Prototype formulations of risperidone-loaded polymeric micelles were evaluated with a largely extended possibility to be able to be administered via the nasal administration route.

## Figures and Tables

**Table 1:** The investigated factors at 3 levels in the optimization process of RIS-loaded polymeric micelles.

Independent Factors	Levels		
	−1	0	+1
RIS amount (mg)	5.0	10.0	15.0
Combined amount of F127 and F108 (mg)	50.0	75.0	100.0

**Table 2:** Main characteristics of the investigated model nasal liquid formulations.

	RIS_PM	RIS-PM_Chit	RIS-PM_HyA
Solvent	pH 5.6 SNES	0.25% *w*/*v* chitosan solution in pH 5.0 purified water	0.50% *w*/*v* HyA solution in pH 5.6 SNES
pH	5.69 ± 0.02	5.04 ± 0.04	5.73 ± 0.07
Viscosity (cP)	1.45 ± 0.01	3.98 ± 0.01	18.5 ± 0.15
Micelle size at 36.5 °C (nm)	22.3 ± 2.1	21.4 ± 0.87	22.7 ± 1.41
Micelle size distribution at 36.5 °C	0.078 ± 0.005	0.142 ± 0.007	0.106 ± 0.010

**Table 3:** Quality Target Product Profile (QTPP) elements and their targets.

QTPP Element	Target
Dosage form	Solution for nasal administration
Nanocarrier	Thermoresponsive polymeric micelles with a size of 10 to 200 nm in monodisperse distribution
Indication	Schizophrenia, bipolar disorders (based on this specific API)
Target population	Mainly adults
Administration route	Nasal
Drug release profile	Rapid and enhanced release
Drug permeation profile	Rapid and efficient permeation

**Table 4:** Results of the 2^3^-factorial design. Data were recorded at 36.5 °C. Data are presented as means ± SD (*n* = 3).

Run No.	Independent Variables		Dependent Variables	
	RIS Amount (mg)	Mixed Co-Polymer Amount (mg)	Micelle Size (nm)	Polydispersity Index
1	5.0	50.0	21.57 ± 2.5	0.312 ± 0.011
2	5.0	75.0	22.37 ± 3.4	0.217 ± 0.009
3	5.0	100.0	21.15 ± 0.8	0.098 ± 0.007
4	10.0	50.0	178.6 ± 11.5	0.276 ± 0.026
5	10.0	75.0	115.6 ± 8.3	0.321 ± 0.044
6	10.0	100.0	97.5 ± 3.5	0.347 ± 0.015
7	15.0	50.0	254.9 ± 8.1	0.485 ± 0.021
8	15.0	75.0	276.2 ± 18.4	0.366 ± 0.016
9	15.0	100.0	201.9 ± 1.4	0.409 ± 0.012

**Table 5:** The effect of pH and viscosity of the LCST. Data are presented as means ± SD (*n* = 3).

pH	LCST (°C)	Viscosity (cP)	LCST (°C)
1	28.1 ± 0.2	5	28.3 ± 0.1
2	28.3 ± 0.1	10	28.3 ± 0.3
3	27.9 ± 0.5	15	28.4 ± 0.3
4	28.4 ± 0.1	20	28.1 ± 0.4
5	28.3 ± 0.2	25	28.1 ± 0.3
6	28.1 ± 0.4	30	28.4 ± 0.1
7	28.5 ± 0.2	35	28.2 ± 0.2
8	28.3 ± 0.3	40	28.4 ± 0.4
9	28.4 ± 0.4		
10	28.2 ± 0.1		
11	28.4 ± 0.3		
12	28.0 ± 0.2		
13	28.2 ± 0.1		
14	28.3 ± 0.2		

## Data Availability

Data are available upon request from the authors.
